# Prediction of Gene Phenotypes Based on GO and KEGG Pathway Enrichment Scores

**DOI:** 10.1155/2013/870795

**Published:** 2013-11-07

**Authors:** Tao Zhang, Min Jiang, Lei Chen, Bing Niu, Yudong Cai

**Affiliations:** ^1^Institute of Systems Biology, Shanghai University, 99 ShangDa Road, Shanghai 200444, China; ^2^State Key Laboratory of Medical Genomics, Institute of Health Sciences, Shanghai Jiaotong University School of Medicine and Shanghai Institutes for Biological Sciences, Chinese Academy of Sciences, Shanghai 200025, China; ^3^College of Information Engineering, Shanghai Maritime University, Shanghai 201306, China; ^4^College of Life Science, Shanghai University, 99 ShangDa Road, Shanghai 200444, China

## Abstract

Observing what phenotype the overexpression or knockdown of gene can cause is the basic method of investigating gene functions. Many advanced biotechnologies, such as RNAi, were developed to study the gene phenotype. But there are still many limitations. Besides the time and cost, the knockdown of some gene may be lethal which makes the observation of other phenotypes impossible. Due to ethical and technological reasons, the knockdown of genes in complex species, such as mammal, is extremely difficult. Thus, we proposed a new sequence-based computational method called *k*NNA-based method for gene phenotypes prediction. Different to the traditional sequence-based computational method, our method regards the multiphenotype as a whole network which can rank the possible phenotypes associated with the query protein and shows a more comprehensive view of the protein's biological effects. According to the prediction result of yeast, we also find some more related features, including GO and KEGG information, which are making more contributions in identifying protein phenotypes. This method can be applied in gene phenotype prediction in other species.

## 1. Introduction

 Recognition of gene phenotypes of proteins is a central challenge of the modern genetics to modulate protein functions and biological processes, and many well-known diseases, such as HIV [1–4], cancers [5–8], chronic liver diseases [9], and Gaucher disease [10], are all closed to protein phenotypes. Hence, determination of protein's phenotypes is quite fundamental and essential in systems biology and proteomics. Except for phenotypes attributes, there are also many other multilabel attributes of proteins, such as subcellular locations [11–13] and multiple functional types of antimicrobial peptides. Multilabel molecule biosystems are very common. 

 During the past decades, numerous efforts have been made in the prediction of gene phenotype of yeast protein based on the following approaches: experimental methods and computational methods. As for experimental approaches, the high-throughput phenotype assays [14, 15] combining with gene perturbation technology [16, 17] provide fast identification for active gene in a response [18]. For example, using yeast mutant strain collections identifies the phenotypes [19]. However, due to the high complexity of phenotypes, it is both costly and time-consuming to determine protein phenotypes by experiments. Sometimes, the results derived from experiment are even of high false rates [20]. Computational methods provide important complementary tools for this problem. Many studies based on sequence-based methods and network-based methods have been made in protein's gene phenotypes identification [21–23]. In this research, we presented a new sequence-based method called *k*NNA-based method to predict gene phenotypes.

## 2. Materals and Methods

### 2.1. Benchmark Dataset

 In this study, 6,732 proteins of yeast were taken from CYGD (the MIPS Comprehensive Yeast Genome Database [24], which collects information on the molecular structure and functional network of the budding yeast. After removing those without sequences, information, or phenotype annotations, the remaining 1,462 composed the benchmark dataset *S*. According to their phenotypes, these proteins were classified into the following 11 categories: (I) conditional phenotypes, (II) cell cycle defects, (III) mating and sporulation defects, (IV) auxotrophies, carbon and nitrogen utilization defects, (V) cell morphology and organelle mutants, (VI) stress response defects, (VII) carbohydrate and lipid biosynthesis, (VIII) nucleic acid metabolism defects, (IX) sensitivity to amino acid anaglogs and other drugs, (X) sensitivity to antibiotics. (XI) sensitivity to immunosuppressants. Let us use *T*_1_, *T*_2_,…, *T*_11_ to represent the tags of the 11 phenotypic categories, where *T*_1_ denotes “conditional phenotypes,” *T*_2_ denotes “cell cycle defects,” and so forth (see column 1 and 2 of Table 1 for the correspondence of tags and phenotypic categories). Thus, the benchmark dataset *S* can be formulated as

(1) $$\begin{array}{c} S=S_{1}\cup S_{2}\cup \cdots \cup S_{11}, \end{array}$$

where *S*_*i*_ represents the set of proteins with tag *T*_*i*_. The IDs of proteins in each *S*_*i*_ are available online in Supplementary Material at http://dx.doi.org/10.1155/2013/870795. From Table 1, we can see that the total number of proteins in each category is much larger than the total number of proteins investigated in this study, this means that some proteins are associated with multiple phenotypes. Like the cases in dealing with the proteins or compounds with multiple attributes [25–29], the proposed method could predict multiclassification phenotypes.

### 2.2. Feature Construction

 The first important step to build an efficient prediction model is to encode each sample by numeric vector. Here, to catch the information of protein phenotype, Gene Ontology (GO) and KEGG enrichment scores were employed to represent the protein, which have been used in some biological problems [30, 31]. Their detailed definition can be found at [30, 31].

### 2.3. Protein Representation and Feature Reduction

 Each protein was represented with 4682 features which include 4583 GO enrichment scores and 99 KEGG enrichment scores. However, among the 4,682 features, some features were with little relationship to the target, which may bring noises to the prediction model. Therefore, these features should be removed. Before removing the irrelevant features, the following formula was used to adjust all features to a standard scale:

(2) $$\begin{array}{c} U_{ij}=\frac{\left(u_{ij}-u_{j}\right)}{T_{j}}, \end{array}$$

where *T*_*j*_ and *u*_*j*_ are the standard deviation and mean value of the *j*th feature, while *u*_*ij*_ and *U*_*ij*_ are the original value and standardized value of the *i*th sample on the *j*th feature. 

 After the transformation, the correlation coefficient between each feature with the target vector was computed and those with correlation coefficient less than 0.1 were discarded. Finally, 989 features remained. Within these 989 features, there were 947 Gene Ontology (GO) enrichment scores and 42 KEGG enrichment scores. Thus, each protein *P*_*z*_ was finally represented by a 989-D vector.

### 2.4. mRMR Method

Minimum Redundancy Maximum Relevance (mRMR), first proposed by Peng et al. [32], is an effective algorithm to identify discriminative features. The detailed algorithm of mRMR can be found at [32] and its program can be downloaded from http://penglab.janelia.org/proj/mRMR/.

mRMR has been widely used in the areas of bioinformatics [25, 33–36].

### 2.5. Prediction Model

#### 2.5.1. *k*NNA-Based Method

 Nearest neighbor algorithm is effective in solving classification and optimization problems in the field of bioinformatics due to its simplicity. It is adopted here to construct the multilabel prediction classifier.

 Within *k*-NNA method, we used the cosine of the angle between two vectors to measure the similarity between them as follows:

(3) $$\begin{array}{c} \mathrm{Cos}\langle p_{x},p_{y}\rangle =\frac{\vec{p_{x}}\cdot \vec{p_{y}}}{||\vec{p_{x}}||\cdot ||\vec{p_{y}}||}, \end{array}$$

where $\vec{p_{x}}\cdot \vec{p_{y}}$ represents the inner product between the *n*-dimensional vector of protein *p*_*x*_ and *p*_*y*_ and ||*p*|| is the modulus of the vector. 

For a query protein, *k* proteins in the training set which are closest to the query protein are first identified and are denoted by *p*_1_, *p*_2_,…, *p*_*k*_. Then, the categories of the query protein can be inferred from the categories of the *k* nearest proteins identified. The procedure of the methodology is described in detail as follows.

(a) Identifying the *k* nearest neighbors of the query protein, denoted by *p*_1_, *p*_2_,…, *p*_*k*_, with the *k* cosines of angle values as *w*_1_, *w*_2_,…, *w*_*k*_.
(b) Then, the following formula:
  (4) $$\begin{array}{c} S\left(P\Rightarrow j\right)=\sum_{i=1}^{k}w_{i}\cdot t_{p_{i},j}\;\left(j=1,2,\ldots ,11\right) \end{array}$$
  is used to calculate the probability that the query protein *P* belongs to the *j*th category, where *t*_*p*_*i*_,*j*_ is the item in *t*_*p*_*i*__ of protein *p*_*i*_. The probabilities (the scores of the 11 categories) calculated above are sorted in descending order for each query protein as
  (5) $$\begin{array}{c} D^{↓}\left\{S\left(P_{z}\Rightarrow j\right)\;|\;j=1,2,\ldots ,11\right\}=V=\left[\begin{array}{c} \begin{array}{c} \begin{array}{c} \mu_{1}\\ \mu_{2} \end{array}\\ \vdots \end{array}\\ \mu_{j}\\ \begin{array}{c} \vdots \\ \mu_{10}\\ \mu_{11} \end{array} \end{array}\right]. \end{array}$$
(c) The corresponding category labels of the category scores are denoted as
  (6) PD↓=[Pμ1,Pμ2,…,Pμi,…,Pμ11]     (i=1,2,…,11),
  where *P* ^*μ*_*i*_^ is the class that scores *i*th in *D* ^↓^.

#### 2.5.2. Comparison with RPC-Based Method

 In the ranking by pairwise comparison (RPC) method, for each pair of labels, a data is allocated to the pair of labels if the data belong to one and only one of the two labels (not both). Given *q* category labels, because there are *C*_*q*_^2^ = *q* · (*q* − 1)/2 possible pairwise combinations of the labels, data subsets, each for corresponding pairwise labels discrimination, are generated.

 Given a new instance, all pairwise classifiers are trained to predict its label, and the ranking of the labels is obtained by counting the votes of each label, where if the instance is classified into a label, the label receives one vote. 

Each dataset contains those examples of *D* that are annotated by at least one of the two corresponding labels, but not both. A binary classifier that learns to discriminate between the two labels is trained from each of these data sets. Given a new instance, all binary classifiers are invoked, and ranking is obtained by counting the votes received by each label.

### 2.6. Evaluation

*(a) Jackknife Testing*. Three methods are often used to evaluate a prediction model, including (1) independent test dataset, (2) subsampling (*K*-fold) test, and (3) jackknife Test. The first method uses unseen data for testing, which needs a large quantity of data. The second method partitions the training set into *k* portions, then taking each portion of the data as the test data and the others (*k* − 1) as the training data. The third one, also named as leave-one-out method, leaves each sample out in turn as the test data and others as the training data. To maximize the quantity of the training data, jackknife test is used to test the predictor developed in the paper; that is, each protein is in turn knocked out as the query protein, and the remaining ones as the training data of the *k*NNA-based method.

*(b) Metric.* Let us define *t*_*z*,*P*_*z*_^*μ*_*i*_^_ = 1 as protein *P*_*z*_ being correctly predicted to class *p*_*z*_^*μ*_1_^; otherwise, *t*_*z*,*P*_*z*_^*μ*_*i*_^_ = 0.

The *i*th prediction accuracy *A* ^*i*^ is calculated as follows (the *i*th order predictions in *P* ^*D*^↓^^):

(7) $$\begin{array}{c} A^{i}=\frac{\sum_{j=1}^{m}t_{j,p_{j}^{\mu_{i}}}}{m}, \end{array}$$

where *m* is the number of the training data. 

### 2.7. Incremental Feature Selection

 Incremental feature selection (IFS) is often used to search out an optimal feature subset that performs best. Specifically, features in the ranked feature set are added one by one from higher to lower rank and the first *n* features that perform best are regarded as the optimal features. When one feature is added, a new feature subset is constructed. Thus, given *N* features, *N* feature subsets will be constructed, where the *i*th -order feature subset is

(8) $$\begin{array}{c} S_{i}=\left\{f_{1},f_{2},\ldots ,f_{i}\right\}\;\left(1\leq i\leq 989\right), \end{array}$$

in which *f*_*i*_ represents the *i*th feature taken from the mRMR ranking.

Each feature subset is used to make prediction and the feature subset (first *n* features) that performs best is deemed as the optimal feature subset. 

## 3. Results and Discussion

### 3.1. Results

#### 3.1.1. mRMR Results

We apply mRMR method to the dataset, and obtain two tables for the features (see Supplementary Material). One is called MaxRel feature table that ranks the features based on their relevance to the class of samples and the other is called mRMR feature table that lists the ranked features by the maximum relevance and minimum redundancy to the class of samples. Such list of ranked features was to be used in the following IFS procedure for the optimal features set selection.

#### 3.1.2. Performance of *k*NNA-Based Method

**Figure 1:**
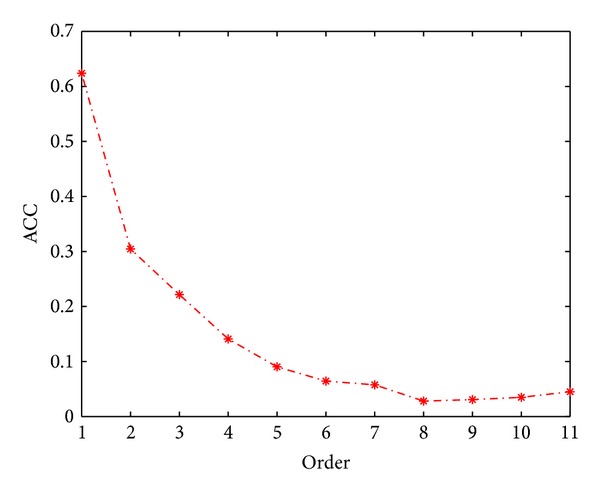
The curve showing the trend of the 11 order prediction accuracies.

**Figure 2:**
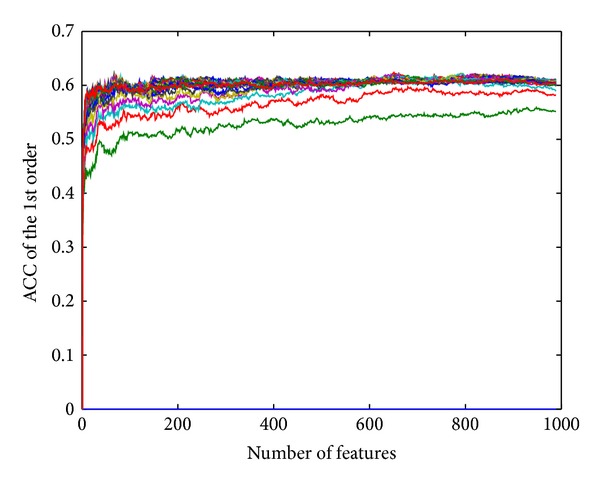
30 IFS curves of *k*NNA-based method corresponding to different values of *k*.

**Figure 3:**
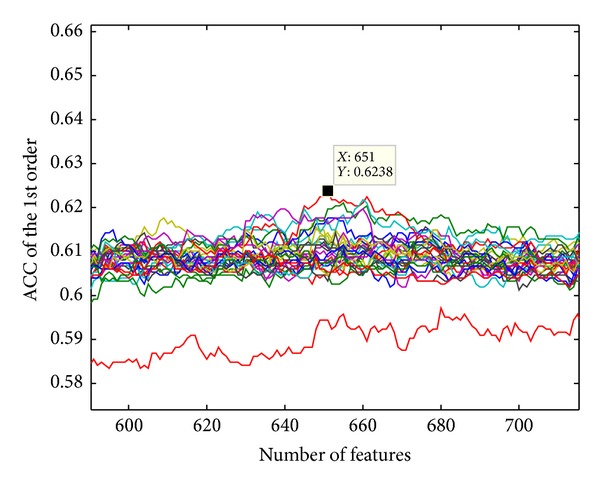
The peak and its coordinate of these IFS curves.

 The first-order prediction accuracy of Jackknife test is 62.38%, while *k* = 17 (*k*-NN) and *n* = 651 (number of optimal features). More details of the 11 order prediction accuracies by using *k*NNA-based method are listed in Table 2 and Figure 1. IFS curve of *k*NNA-based method can be seen in Figure 2, which contains 30 curves corresponding to different values of *k*, and their detailed computing results of accuracy (ACC) can be seen at Supplementary Material. We highlighted the peak area of these curves to find optimal *k* in Figure 3.

#### 3.1.3. Performance of RPC-Based Method

 Firstly, we classify the total labels into 55(*C*_11_^5^) sublabels. Select the sample which meets the demands that one sample belongs to one and only one of the two labels (not both). Then, 55 binary subsets were constructed. Three well-known binary classification algorithms including RandomForest, SMO, and Dagging were applied to build the prediction model. The prediction results are summarized in Table 3. 

#### 3.1.4. Comparison with RPC-Based Method

We compared the first-order prediction accuracy of our method with the first-order prediction accuracy of RPC-based method. It can be found that the first-order prediction accuracies of RPC-based method using Dagging, RandomForest, and SMO are all lower than our *k*NNA-based method. 

### 3.2. Discussion

 To illustrate the biological meanings of the selected optimal feature subset, we firstly classified GO terms into three kinds: the biological process, cellular component, and molecular function GO terms. The 622 GO terms in the mRMR feature list were mapped to the Gene Ontology (GO) terms, the children of the three root GO terms. The figures show the frequency of each GO term in the feature subset, and display the ratio of the number of each GO term to the scale of the number of its children terms.

#### 3.2.1. Biological Process GO Terms

 In BP frequency, the top five GO biological process terms are GO:0009987: cellular process (399), GO:0008152:  metabolic process (316), GO:0019740: nitrogen utilization (216), GO:0065007: biological regulation (136), and GO:0050789: regulation of biological process (131). In BP percentage, the top five GO biological processes are GO:0019740: nitrogen utilization (4.20%), GO:0071840: cellular component organization or biogene (3.57%), GO:0000003: reproduction (2.94%), GO:0022414: reproductive process (2.88%), and GO:0009987: cellular process (2.04%). For both GO biological process term number and percentage distribution analysis, the GO terms corresponding to the nitrogen utilization (GO:0019740) and cellular process (GO:0009987) were highlighted within the top five GO terms. This indicates that proteins assigned with these two GO terms may affect protein phenotype determination greatly. This conclusion is consistent with the common knowledge that specific cellular biological activities of the proteins confer with special phenotypes. It was also reported by Granek and Magwene that two key signaling networks: the filamentous growth MAP kinase cascade and the Ras-cAMP-PKA pathway, can regulate the yeast colony morphology response [37]. Additionally, the yeast cell wall integrity pathway was involved in resistance of the yeast *Saccharomyces cerevisiae *to the biocide polyhexamethylene biguanide [38]. 

The highlight of nitrogen utilization (GO:0019740) suggests that the nitrogen utilization, which is essential for life survival and development, may have more definite affection on protein phenotype. Nutrient stresses trigger a variety of developmental switches in the budding yeast *Saccharomyces cerevisiae*. It was demonstrated that low levels of carbon combined with abundant nitrogen trigger complex colony formation in yeast [37].

#### 3.2.2. Cellular Component GO Terms

In CC frequency, the top six GO cellular component terms are GO:0005623: cell (171), GO:0044464: cell part (169), GO:0043226: organelle (135), GO:0044422: organelle part (103), GO:0032991: macromolecular complex (84), and GO:0031974: membrane-enclosed lumen (39). In CC percentage, the top six GO cellular component terms are GO:0031974: membrane-enclosed lumen (12.4%), GO:0044422: organelle part (8.42%), GO:0043226: organelle (8.4%), GO:0032991: macromolecular complex (5.20%), GO:0044464: cell part (4.77%), and GO:0005623: cell (4.20%). For both GO cellular component term number and percentage distribution analysis, the GO terms corresponding to the organelle (GO:0043226) and organelle part (GO:0044422) were highlighted within the top six GO terms. It may be concluded that proteins located in all cellular organelles should be guaranteed. It suggests that organelles, which have specific structural and functional attributes, may possess more definite protein phenotype to carry out their specific functions. This also implicated that proteins assigned to these GO terms could contribute relatively more to the overall protein phenotype determination. For example, the communication between mitochondrial and nuclear loci (i.e., *COX1*-*MSY1* and *Q0182*-*RSM7*) showed significant reductions in the absence of mitochondrial encoded reverse transcriptase machinery [39]. The inclusion of macromolecular complex (GO:0032991) suggests that proteins expressing some phenotype need to interact with each other to function together and that macromolecular complex should certainly determine the phenotype of proteins. The inclusion of membrane-enclosed lumen (GO:0031974) also suggests that proteins assigned to this cellular component could greatly contribute to protein phenotype, because most of the cellular organelles are enclosed by membrane, such as mitochondrial and nucleus.

#### 3.2.3. Molecular Function GO Terms

In MF frequency, the top six GO molecular function terms are GO:0003824: catalytic activity (79), GO:0005488: binding (69), GO : 0001071: nucleic acid binding transcription factor activity (40), GO:0000988: protein binding transcription factor activity (14). GO:0065009: regulation of molecular function (8), and GO:0005215: transporter activity (7). Proteins assigned to these three GO terms required binding or interaction to carry out their structural or functional activities. This suggests that proteins assigned to these six GO terms contributed profoundly to the protein phenotype. In MF percentage, the top six GO molecular function terms are GO:0009055: electron carrier activity (25%), GO:0016530: metallochaperone activity (25%), GO:0045182: translation regulator activity (14.3%), GO:0005198: structural molecule activity (11.8%), GO:0001071: nucleic acid binding transcription factor activity (9.0%), GO:0005488: binding (3.99%), and GO:0016209: antioxidant activity (3.85%). The relatively small base number made protein GO terms influencing protein phenotype relatively more enriched in the top six molecular function GO terms, especially in electron carrier activity (GO:0009055) and metallochaperone activity  (GO:0016530). The highlight of electron carrier activity (GO:0009055) may be attributed to the relatively limited and definite function of these proteins. It was reported that some ontology drug can interact with the electron transport chain (ETC) to generate high levels of ROS within the organelle and consequently cell leads to death [40]. The highlight of metallochaperone activity (GO:0016530) may be ascribed to that metalloprotein used to express specific function with metallochaperone and metallic ion. In all bacteria, a panel of metalloregulatory proteins controls the expression of genes encoding membrane transporters and metal trafficking proteins [41]. Because of the large base number of the top six GO terms in MF frequency, they have relatively lower enrichment within the top eight GO terms in MF percentage. 

## Supplementary Material

Supplementary Material 1*：*The ID of yeast 1462 proteins with phenotype annotation.
Supplementary Material 2*：*The MaxRel and mRMR feature tables using mRMR method.
Supplementary Material 3: The 11 order prediction accuracies based on mRMR features list using kNNA-based method (k=1,2, ...31).

## Figures and Tables

**Table 1:** Breakdown of 1462 budding yeast proteins according to their 11 phenotypes.

Tag	Phenotype category	Number of proteins
*T*_1_	Conditional phenotypes	536
*T*_2_	Cell cycle defects	272
*T*_3_	Mating and sporulation defects	198
*T*_4_	Auxotrophies, carbon, and nitrogen utilization defects	266
*T*_5_	Cell morphology and organelle mutants	535
*T*_6_	Stress response defects	147
*T*_7_	Carbohydrate and lipid biosynthesis	46
*T*_8_	Nucleic acid metabolism defects	219
*T*_9_	Sensitivity to amino acid analogs and other drugs	124
*T*_10_	Sensitivity to antibiotics	43
*T*_11_	Sensitivity to immunosuppressants	14

Total	—	2,400

**Table 2:** The 11 order prediction accuracies by *k*NNA-based method.

	Method order										
	1	2	3	4	5	6	7	8	9	10	11
*k*NN-based method (ACC)	62.38	30.44	22.16	14.09	9.03	6.43	5.75	2.8	3.08	3.49	4.51

**Table 3:** The 11 order prediction accuracies by RPC-based methods (Dagging, RandomForest, SMO).

	Methods order										
	1	2	3	4	5	6	7	8	9	10	11
Dagging	60.05	33.58	21.96	13.75	10.53	8.28	6.57	3.56	2.6	1.85	1.44
RandomForest	58.62	34.2	22.3	14.7	9.92	7.66	5.95	5.2	3.28	1.5	0.82
SMO	56.16	34.68	21.55	14.84	10.88	7.8	6.36	4.65	3.21	2.26	1.78
